# A Limited Sampling, Simple, and Useful Method for Determination of Glomerular Filtration Rate in Cats by Using a New Accurate HPLC Method to Measure Iohexol Plasmatic Concentrations

**DOI:** 10.1155/2013/569121

**Published:** 2013-11-12

**Authors:** Meucci Valentina, Guidi Grazia, Melanie Pierre, Breghi Gloria, Lippi Ilaria

**Affiliations:** Department of Veterinary Clinics, University of Pisa, San Piero a Grado, Via Livornese Lato Monte, 56122 Pisa, Italy

## Abstract

Glomerular filtration rate (GFR) is still a highly underutilized tool in cats because available methods are not easy to be performed in clinical practice. Iohexol (IOX) has been shown to be a useful and reliable marker of GFR both in animals and in humans. The aim of the present study was to develop a rapid and reliable method for measuring IOX in feline plasma and to evaluate the accuracy of limited sampling models to establish a low-cost and clinically suitable GFR test. IOX concentrations were determined by using a new HPLC-UV method. GFR was assessed as plasma clearance of IOX, which was calculated by dividing dose administered by area under the curve of plasmatic concentration *versus* time (AUC), and indexed to body weight (BW). Correlation and agreement analysis between the GFR values obtained by a seven-point clearance method and the GFR values determined by the application of simplified sample combinations indicated that the 3-blood sample clearance model (5, 30, and 60 min) was the best simplified method because it provided an accurate GFR value in only one hour. The reported method is a simple and accurate way of GFR determination, which may be easily used in a clinical setting.

## 1. Introduction

Chronic kidney disease (CKD) is one of the most common disorders in cats, especially in older ones for which it represents a major cause of illness and death [1, 2]. Plasma creatinine (PCr) and urea (PU), the most used parameters for assessing renal function in veterinary practice, cannot be used in the early diagnosis of renal failure because they start to rise too late, when the 75% of functioning nephrons are lost [3, 4], and are also affected by several extra renal factors [5]. Glomerular filtration rate (GFR), directly related to the functional renal mass, is considered the most sensitive and early marker of kidney failure [6]. At present, in veterinary medicine, GFR can be assessed by using different methods, which have both advantages and disadvantages. Many methods have several disadvantages including the labour intense nature, risks caused by anaesthesia, cost of the test substance, assay of the substance used, or need for specialized licensing and equipment. The traditional gold standard for measurement of GFR (urinary clearance of inulin) is not suitable in a clinical setting [7, 8]. These methods are labor intensive and require the placement of an indwelling urinary catheter with associated risk of sedation and of causing lower urinary tract infection. Over the past two decades, many alternative methods for determining GFR have been shown to provide acceptable measurements of renal function in cats [9]. Unfortunately, most of these methods are not routinely available for use by veterinary practitioners in a clinical setting due to lack of drug availability (inulin, exogenous creatinine) or the need for special licensing and equipment (radiolabeled compounds) [10–13]. Plasma clearance of iohexol (IOX) has been shown to provide a reliable estimate of GFR in cats [9, 14–16]. More recently, limited sampling strategies were also investigated in order to estimate GFR by the use of colorimetric assays or correction formula [17–19]. In feline patients, the use of three-sample and one-sample procedures through plasma clearance of iodixanol showed a close correlation with multisample inulin method in both clinically healthy and CKD cats [20]. A single sample at 180′ after-injection using corrected slope-intercept IOX clearance seemed to provide an accurate estimation of GFR in cats, although further investigations are still required for very low or very high values of GFR [18]. Nevertheless, there is a need of simplification of assay GFR protocols by validating detection methods of markers and reducing sampling times [21]. It is also important that each laboratory establishes its own reference range for a given GFR protocol in several animal species. Plasma concentration of IOX can be detected indirectly by measuring plasma levels of iodine or directly by the use of several methods including X-ray fluorescence [22, 23], inductively coupled plasma atomic emission spectroscopy [24], high-performance liquid chromatography (HPLC) [25–27], colorimetric assay [5, 13–28], and capillary electrophoresis [29]. However, HPLC is the most widely used technique for analysis of IOX in clinical practice due to its sensitivity and flexibility [30, 31]. In urine, plasma, and serum, IOX is present as two isomers, called endo- and exoiohexol both of which can be used for HPLC quantification and GFR measurement [25–31].

The aims of the present study were to develop a rapid and reliable HPLC analysis method for measuring IOX in feline plasma and to evaluate the accuracy of limited sampling models to establish an accurate, low-cost, and clinically suitable GFR test in cats.

## 2. Materials and Methods

### 2.1. Chemicals and Reagents

IOX and the internal standard (IS) of iopentol were kindly supplied by Nycomed Amersham Sorin (Milan, Italy). Water was doubly distilled and purified using a Sartorius cellulose acetate filter (Göettingen, Germany). HPLC grade water, dichloromethane, and acetonitrile were supplied by LABSCAN (Hasselt, Belgium).

### 2.2. Chromatographic Conditions

The HPLC system consisted of a Series 200 Perkin Elmer gradient Pump coupled to a Series 200 Perkin Elmer variable UV detector which was set at 254 nm. The reversed-phase column was a SunFire Waters C_18_ column (5 *μ*m, 250 × 4.60 mm) connected to a Waters Guard-Pak C_18_ precolumn (4 *μ*m) (Waters, Milford, MA, USA). The column was kept at room temperature. Turbochrom software was used for data processing. A 20 *μ*L injection of sample was used each time. The mobile phase consisted in acetonitrile-water pH 2.7 (acidified by addition of H_3_PO_4_ 85%). Both IOX and IS were eluted as two isomers. For analysis, the peak area of the major IOX and IS isomers was used because these constituted more than 80% of the combined peak areas and the ratio of both the isomer peaks remained constant at different IOX and IS concentrations under the current analytical condition. All calculations were performed using peak area ratios of the larger IOX peak to the IS peak (peak area ratio) by the use of Microsoft Excel (MS OFFICE, 2007).

### 2.3. Preparation of Stock Solutions

Stock solutions of IOX and IS were prepared monthly as 1 mg/mL solutions in double-distilled water and stored at −20°C. IOX working solutions were made by further diluting the stock solutions and were prepared fresh daily. A total of seven concentrations of IOX including 2.5, 10, 25, 50, 125, 250, and 500 *μ*g/mL in drug free feline plasma were used as calibrators. Three in-house quality control standards containing IOX at low (25 *μ*g/mL), medium (125 *μ*g/mL), and high (500 *μ*g/mL) concentrations were also prepared in feline plasma and were used for assay validation. Aliquots of the IS stock solution were diluted in water to produce a working (50 *μ*g/mL) IS solution. Aliquots of the calibrators, quality control samples, and reference standard solutions were stored at −20°C until use. A total of nine standard curves were prepared and all calibrators or quality control samples were injected in triplicate.

### 2.4. Validation

The HPLC method was validated according to international rules [32]: specificity, sensitivity, linearity, limits of determination (LOD) and of quantification (LOQ), repeatability, and reproducibility were determined. For the linearity test calibration curves with IOX working as standard solutions at 0.5–100 *μ*g/mL in water were prepared. Feline plasma samples spiked with IOX at 2.5, 10, 25, 50, 125, 250, and 500 *μ*g/mL were analyzed using the HPLC method. Taking into account dilution step, these spiked samples corresponded to IOX standard concentrations of 0.5, 2, 5, 10, 25, 50, and 100 *μ*g/mL. The experiment was repeated nine times. To evaluate specificity, blank samples of feline plasma containing no IOX or IS were analyzed to check for the presence of interfering peaks at the elution time of IOX and IS. The repeatability was tested by analyzing samples of feline plasma spiked with IOX (*n* = 63). Samples were spiked at the levels of 25 *μ*g/mL (corresponding to 5 *μ*g/mL), 125 *μ*g/mL (corresponding to 25 *μ*g/mL), and 500 *μ*g/mL (corresponding to 50 *μ*g/mL). All samples were measured in triplicate on the same day. For the within-laboratory reproducibility test, each spiked level was tested in triplicate over seven days. The results of these experiments were used also for the determination of the recovery. The analytical recovery of IOX was assessed by comparing the peak area ratio of spiked samples with the peak area ratio (analyte peak area/IOP peak area) of the reference standards prepared in water. The sensitivity of the method was expressed as the LOQ, which is the minimum concentration of IOX in plasma that can be quantitatively determined with a peak height to baseline ratio of at least 10 : 1, and the LOD, which has a peak height to baseline ratio of 3 : 1. To evaluate stability, aliquots of spiked samples were subjected to three cycles of freeze and thaw (freezing for 24 h at −20°C and thawing unassistedly at room temperature). For short-term stability test, the aliquots of the spiked samples were thawed at room temperature and kept at this temperature for 6 h (the duration of analysis for a typical batch) before analysis. For long-term stability test, the aliquots of the spiked samples were thawed at room temperature and kept at this temperature for 12 and 24 h before analysis.

### 2.5. Animal Study

Fifty privately owned domestic short-hair cats, presented to the Department of Veterinary Science for minor surgery and/or neutering, were included in the study after the owner's informed consent and Ethical Committee approval (University of Pisa authorization number 8317). The cats were divided into two groups: nonazotaemic cats (PCr < 141 *μ*mol/L) and azotaemic cats (PCr > 141 *μ*mol/L) according to IRIS guidelines. The body weight of the cats ranged from 2 to 5.5 kg (mean 3.37 ± 0.71 kg) and their age from 1 to 3 years (mean 1.8 ± 0.7 years). All cats were assessed not to be affected by concurrent diseases on the basis of physical examination, complete blood count, plasma biochemical analysis, urinalysis, testing for FeLV and FIV, and abdominal ultrasound. 

Each cat was fasted overnight (at least 12 h) before the experimental procedure, and no food was given during the trial. Water was given ad libitum. A commercially available IOX formulation was administered IV as a bolus (within 1 minute) at the dose of 64.7 mg/kg (0.1 mL/kg) through the right jugular catheter. The syringe and the needle used to infuse IOX were weighed before and after injection, in order to determine the exact administered dose. Samples were collected by the catheter positioned into the left jugular vein before marker's administration (time 0) and at 5, 30, 60, 120, 240, 360, and 480 minutes after the completion of the injection. Blood was collected into lithium heparin test tubes and centrifuged at 3500 rpm within 10 minutes from collection. Plasma was stored in aliquots at −20°C.

### 2.6. Preparation of Plasma Samples

Each plasma sample was submitted to a preparation method for the extraction of IOX. Fifty microliters of plasma was added to a 50 *μ*L water solution of IS (50 *μ*g/mL) and vigorously vortexed (60 seconds). The plasma sample was deproteinized by adding 100 *μ*L of dichloromethane (CH_2_Cl_2_), extracted with double-distilled water (150 *μ*L), vigorously vortexed (60 seconds), and centrifuged at 3500 rpm for 10 minutes. Twenty microliters of the supernatant was centrifuged at 3000 rpm for 10 minutes and then injected into the HPLC system.

### 2.7. Pharmacokinetic and Statistical Analysis

Pharmacokinetic analyses were performed by WinNonlin Version 5.1^a^. Plasma data were subjected to noncompartmental analysis with a statistical moment approach. The area under the plasma concentration *versus* time curve (AUC) was calculated by trapezoidal rule with extrapolation to infinity. Plasma clearance of IOX was determined by dividing dose administered by AUC, and indexed to body weight (BW) (mL/min/kg). The administered dose was established by assuming that the 85% of IOX was exoiohexol. The normalized seven-point clearance value was considered a reference for the evaluation of simplified methods. Correlation and agreement analysis between the GFR values obtained by the seven-point clearance method and the GFR values determined by the application of simplified sample combinations were performed using Pearson test, linear regression analysis, and Bland-Altman plots. In addition, the accuracy of the GFR estimates was determined as the percentage of results not deviating more than 15%, 30%, and 50% from the GFR values obtained by the seven-point clearance method. The percentages between the formulas were compared using the Chi-square test.

For simplified methods GFR was calculated by using the same pharmacokinetic model of the reference method. Among the possible different models, four simplified sample combinations (Models A, B, C, and D) were chosen. Each model showed a different sample combination: Model A (5, 30, 60, and 240 minutes), Model B (5, 30, 60, and 120 minutes), Model C (5, 60, 120, and 240 minutes), and Model D (5, 30, and 60 minutes). Comparisons of nonazotaemic and azotaemic groups of cats were based on Student's *t*-test. A *P* value below 0.05 was considered significant.

## 3. Results

### 3.1. HPLC Method

**Figure 1:**
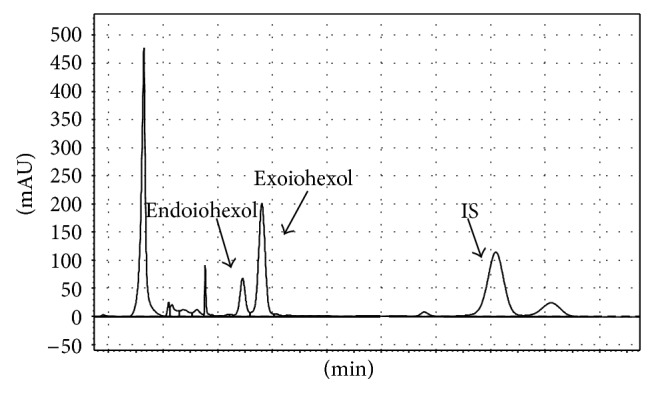
Chromatogram of the separation of IOX (10 *μ*g/mL) and IS extracted from feline plasma; IS: internal standard.

Figure 1 illustrates chromatogram of IOX and IS in extracted feline plasma. IOX was eluted as two isomers endoiohexol and exoiohexol, at 6.4 and 6.8 min, respectively, whereas the IS was eluted as two isomers at 10.4 and 11.0 min. The specificity of the method was tested by analyzing feline plasma samples before the administration of IOX. No interfering peaks were observed at the elution times of IOX or IS isomers. IOX LOD and LOQ were found to be 0.01 and 0.1 *μ*g/mL, respectively. Calibration graphs for IOX (*n* = 9) were constructed over the concentration range of 0.5–500 *μ*g/mL and showed an average correlation coefficient (*R* ^2^) of 0.999. The accuracy of the estimated IOX concentration was more than 90% at three concentrations. The precision expressed as interday coefficient of variation (CV%) ranged from 3.8% to 6.5% and as the intraday CV% ranged from 1.5% to 4.0% (Table 1). The extraction method of IOX from plasma samples had an average recovery ranging from 96.0 ± 2.5% to 95.0 ± 2.1% for low-to-high spiked samples (Table 1). The recovery was reproducible over seven replications performed over 7 different days. IS had an average recovery ranging from 96.0 ± 2.5% to 95.0 ± 1.5%. The concentrations of IOX in freeze-thaw and short-term stability evaluation were not significantly different from the fresh calibrators. The accuracy of the spiked samples ranged from 98% to 100% and from 98% to 101% after the freeze-thaw stability and short-term stability testing, respectively. The formal ruggedness test was conducted when the method was validated on a second HPLC system (Thermo Finnigan, Waltham, MA, USA) by another analyst (results not shown). Using the optimized parameters, the method was found to be equally robust.

### 3.2. Clearance of IOX

**Figure 2:**
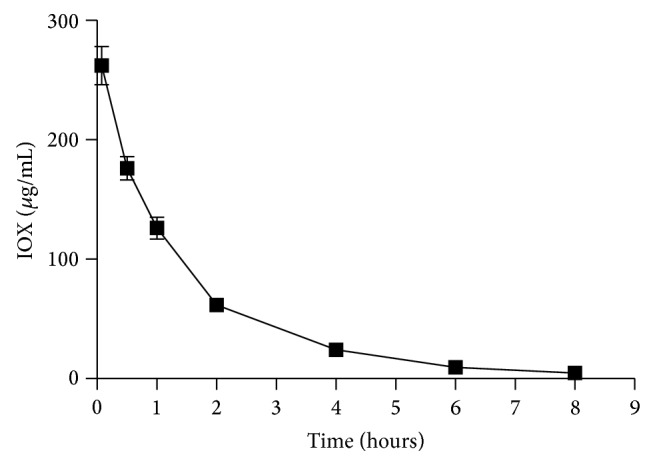
Plasma exoiohexol concentration *versus* time profile from 50 cats after a single administration of IOX (at a nominal dose of 64.7 mg/kg); data are expressed as mean ± standard error bars.

The GFR protocol used was well tolerated in all animals and no adverse effect was noticed or reported by the owner after the test. The plasma concentrations *versus* time profiles for IOX obtained from seven-point clearance method (5, 30, 60, 120, 240, 360, and 480 minutes) in all analyzed cats are reported in Figure 2. The extrapolated part of the AUC in all the analyzed models did not exceed 25% of the total AUC. 

The nonazotaemic group consisted of 35 cats. PCr ranged from a minimum of 78 *μ*mol/L to a maximum of 140 *μ*mol/L (mean: 106 *μ*mol/L). Reference GFR (7-point clearance method) ranged from a minimum of 1.21 mL/min/kg to a maximum of 8.62 mL/min/kg, with a mean value of 3.40 ± 0.29 mL/min/kg. Model A GFR ranged from a minimum of 1.52 mL/min/kg to a maximum of 6.80 mL/min/kg, with a mean value of 3.32 ± 0.47 mL/min/kg. Model B GFR ranged from a minimum of 1.64 mL/min/kg to a maximum of 6.62 mL/min/kg, with a mean value of 3.28 ± 0.38 mL/min/kg. Model C GFR ranged from a minimum of 1.40 mL/min/kg to a maximum of 6.87 mL/min/kg, with a mean value of 3.08 ± 0.41 mL/min/kg. Model D GFR ranged from a minimum of 1.51 mL/min/kg to a maximum of 8.30 mL/min/kg, with a mean value of 3.43 ± 0.39 mL/min/kg.

The azotaemic group consisted of 15 cats with PCr above the reference range (141 *μ*mol/L). PCr ranged from a minimum of 143 *μ*mol/L to a maximum of 209 *μ*mol/L (mean: 160 *μ*mol/L). Reference GFR (7-point clearance method) ranged from a minimum of 1.36 mL/min/kg to a maximum of 3.47 mL/min/kg, with a mean value of 2.38 ± 0.19 mL/min/kg. Model A GFR ranged from a minimum of 1.25 mL/min/kg to a maximum of 2.99 mL/min/kg, with a mean value of 2.11 ± 0.17 mL/min/kg. Model B GFR ranged from a minimum of 1.49 mL/min/kg to a maximum of 4.17 mL/min/kg, with a mean value of 2.57 ± 0.23 mL/min/kg. Model C GFR ranged from a minimum of 1.35 mL/min/kg to a maximum of 3.66 mL/min/kg, with a mean value of 2.28 ± 0.19 mL/min/kg. Model D GFR ranged from a minimum of 1.45 mL/min/kg to a maximum of 3.81 mL/min/kg, with a mean value of 2.26 ± 0.26 mL/min/kg.

*t*-test analysis showed a significant difference in GFR, PCr, and PU values between nonazotaemic and azotaemic cats at *P* = 0.003, *P* < 0.0001, and *P* = 0.0006, respectively (Table 2). *t*-test analysis showed no significant difference in bodyweight values between nonazotaemic and azotaemic cats (*P* > 0.05) (Table 2). 

### 3.3. Correlation Analysis: Nonazotaemic Cats

**Figure 3:**
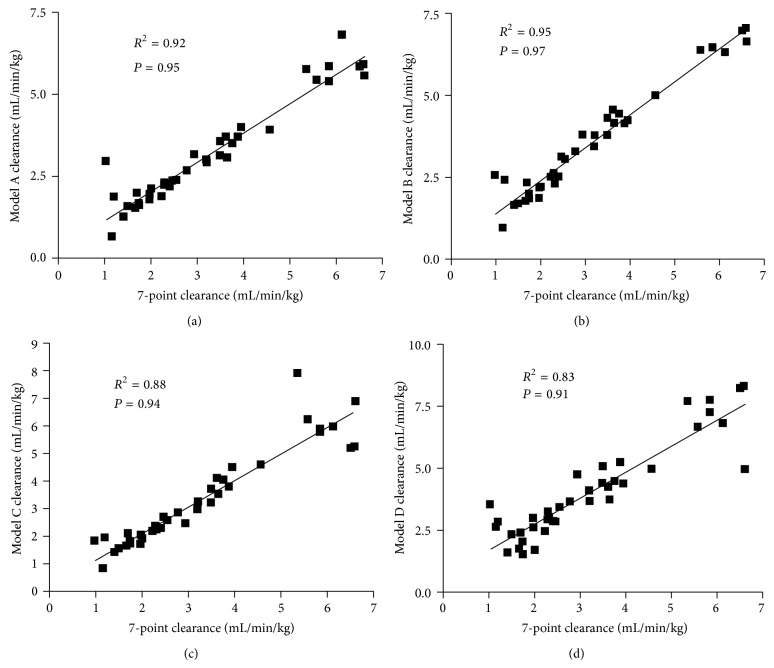
The correlation between the 7-point reference clearance and simplified clearance models (a), (b), (c), and (d) in nonazotaemic cats (*n* = 35).

**Figure 4:**
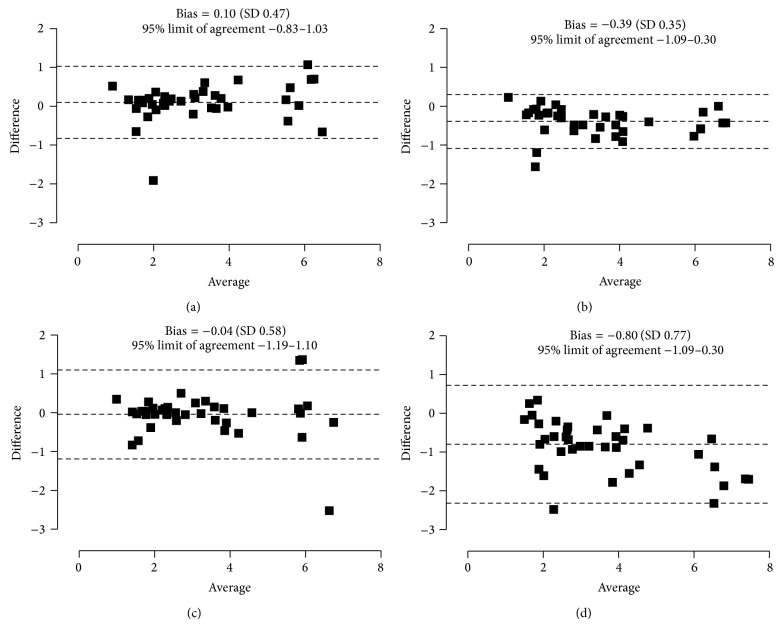
Bland-Altman plots of difference *versus* average of GFR between the 7-point reference clearance and simplified clearance models (a), (b), (c), and (d) in nonazotaemic cats (*n* = 35).

Pearson correlation testing between GFR values obtained by reference method and Model A (5, 30, 60, and 240 mins) showed a positive linear correlation (*R* ^2^ = 0.92, *P* = 0.95; Figure 3(a)). Pearson correlation testing between GFR values obtained by reference method and Model B (5, 30, 60, and 120 mins) showed a positive linear correlation (*R* ^2^ = 0.95, *P* = 0.97; Figure 3(b)). Pearson correlation testing between GFR values obtained by reference method and Model C (5, 60, 120, and 240 mins) showed a positive linear correlation (*R* ^2^ = 0.88, *P* = 0.94; Figure 3(c)). Pearson correlation testing between GFR values obtained by reference method and Model D (5, 30, and 60 mins) showed a positive linear correlation (*R* ^2^ = 0.83, *P* = 0.91; Figure 3(d)). The results from Bland-Altman analysis are given in Figure 4. The accuracies of all models tested were not significantly different from those of the reference method (Table 3) .

### 3.4. Correlation Analysis: Azotaemic Cats

**Figure 5:**
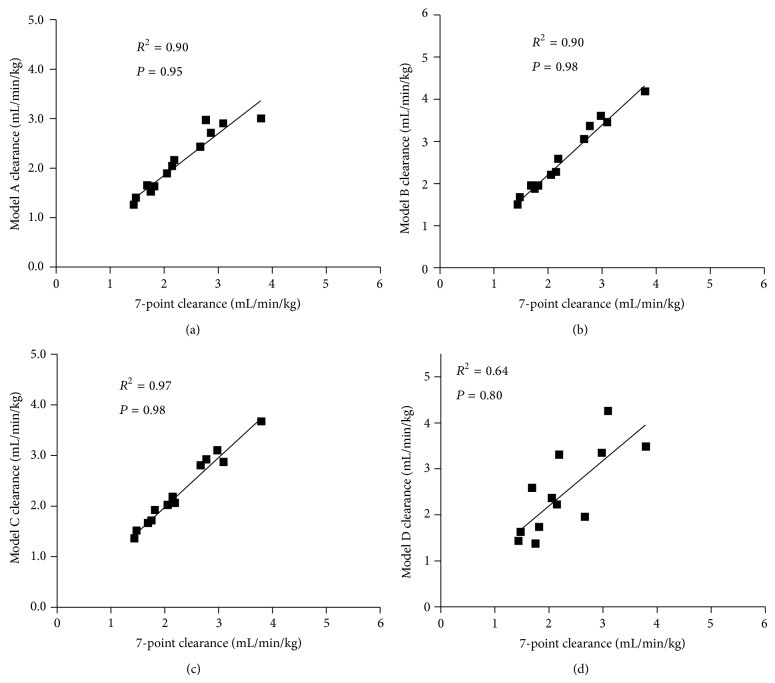
The correlation between the 7-point reference clearance and simplified clearance models (a), (b), (c), and (d) in azotaemic cats (*n* = 15).

**Figure 6:**
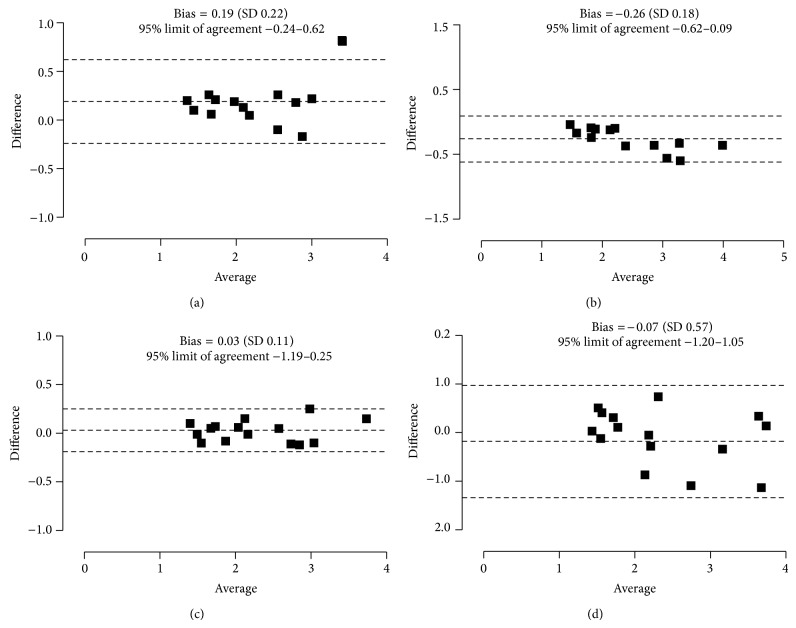
Bland-Altman plots of difference *versus* average of GFR between the 7-point reference clearance and simplified clearance models (a), (b), (c), and (d) in azotaemic cats (*n* = 15).

Pearson correlation testing between GFR values obtained by reference method and Model A (5, 30, 60, and 240 mins) showed a positive linear correlation (*R* ^2^ = 0.90, *P* = 0.95; Figure 5(a)). Pearson correlation testing between GFR values obtained by reference method and Model B (5, 30, 60, and 120 mins) showed a positive linear correlation (*R* ^2^ = 0.97, *P* = 0.98; Figure 5(b)). Pearson correlation testing between GFR values obtained by reference method and Model C (5, 60, 120, and 240 mins) showed a positive linear correlation (*R* ^2^ = 0.97, *P* = 0.98; Figure 5(c)). Pearson correlation testing between GFR values obtained by reference method and Model D (5, 30, and 60 mins) showed a positive linear correlation (*R* ^2^ = 0.64, *P* = 0.80; Figure 5(d)). The results from Bland-Altman analysis are given in Figure 6. The accuracies of all models tested were not significantly different from those of the reference method (Table 3).

Plasma clearance of IOX, determined with 3-point clearance Model D, in analyzed nonazotaemic cats ranged from 1.51 to 8.30 mL/min/kg (mean ± SEM, 4.07 ± 0.34 mL/min/kg) while in azotaemic cats it ranged from 1.45 to 3.81 mL/min/kg (mean ± SEM 2.26 ± 0.21 mL/min/kg). There were significant differences between the two groups in GFR (*P* = 0.008) (Table 2).

## 4. Discussion

The importance of early diagnosis in slowing down the progression of CKD has been widely demonstrated both in veterinary medicine and in human medicine [33–35]. Unfortunately, although GFR is universally considered the gold standard test to evaluate overall renal function, its use in veterinary clinical practice is still uncommon, due to technical difficulties, high number of samples, and low availability of markers.

The present study showed a fast, accurate, and relatively simple method for GFR determination in feline patients by using IOX plasma clearance evaluation. Plasma clearance methods are easier to be performed compared to urinary clearance methods and represent an attractive way of GFR determination. IOX has been shown to be a good alternative to inulin and radioactive tracers in human [36], pig [37, 38], horse [39], donkey [40], dog and cat [13–41].

Currently available HPLC systems to determine plasma IOX concentration have been proven to give reliable data, but they still present some disadvantages to be routinely used for the early diagnosis of CKD in a clinical setting. The method investigated in the present study has combined an easy sample preparation and a rapid HPLC run with a simple mobile phase. Analysis has been performed with inexpensive, nonhazardous, and readily available chemicals. The robustness of the method enables ease for operators to learn the technique and to generate reproducible results. The method indeed is very economical, with an approximate cost per sample of less than two Euros for supplies and materials. In fact, a single analytical column, under the assay condition, has lasted for the entire period of method validation and clinical study. Furthermore, the stability test has indicated that plasma samples can be frozen or sent by mail, and this would be attractive for general practitioners, who could send samples to a reference laboratory. This HPLC method requires a very small volume of plasma (50 *μ*L). Such limited amounts of plasma may be a significant advantage in a feline clinical setting, especially when anaemic or dehydrated animals are involved. At the dosage used in this study, IOX can be safely utilized even in debilitated or severely azotaemic subjects. Furthermore, no one of the enrolled cats has shown immediate or subsequent side effects. In the authors' experience the use of two intravenous catheters (one utilized for IOX injection and the other one for taking blood samples) has increased the compliance of owners and patients.

The use of a seven-sample method to estimate plasma clearance of a tracer is an extremely accurate way of performing curve fitting with nonlinear regression analysis, but it is too time consuming and expensive and it could be excessively stressful for feline patients. Furthermore, the high number (seven) of blood samples required is too cumbersome to be used in a clinical setting. Limited sampling strategies for plasma clearance procedures have been investigated extensively in humans [42] and more recently in animals [43, 44] for establishing a quick, inexpensive, and clinically accurate value of GFR. 

In the present study, plasma clearance values were determined by a noncompartmental approach. This approach is more convenient if the sampling period covers a sufficient period because it does not require specific mathematical modelling. The only parameter required, and which is easily calculated, is the AUC. The dose/AUC approach has shown to be both highly reproducible and the most precise and accurate strategy for GFR determination in healthy humans using sinistrin as marker [45]. It was demonstrated in the same study that a better estimation of GFR was obtained by extrapolating to infinity. The extrapolated part of the AUC should not exceed 20% of the total AUC. In the present study the extrapolated part of the AUC did not exceed 25% with all the simplified models analysed, and this in combination with fast, cheap, and accurate methods of analysis is therefore adequate for GFR estimation in cats in clinical settings. All simplified models, that have been taken into consideration in the present study, have shown a high accuracy in determining GFR, in both nonazotaemic and azotaemic patients, and have been a significant simplification of the reference method, in terms of time and number of samples. Anyway, among different models, Model D has shown the best solution because it has combined an enough accurate GFR determination with a very quick and easy-to-perform method. Model D was chosen as the best simplified method also because it provided an enough accurate and precise GFR value using 3 blood samples only in one hour. This model is not the most precise one among all tested, but it represents a good compromise between precision and owner and patient compliance. An easy and rapid assay to determine GFR would be extremely useful for an early diagnosis of CKD in feline patients.

In conclusion, the present study has validated a safe, simple, and accurate three-sample HPLC method for the determination of GFR through the plasma clearance of IOX in feline patients. This method represents an attractive and cheap alternative to cumbersome plasma clearance methods, with a dramatic applicatory potential in different clinical settings. The accuracy of HPLC analysis, the possibility to mail plasma sample to a referring laboratory, and the high compliance of this method would lead general practitioners to an even more easy diagnosis of subclinical stages of CKD and to a better management of the disease.

## Figures and Tables

**Table 1:** Recovery and intra- and interday precision results for the assay; CV: coefficient of variation; intraday *n* = 9 in one day; interday *n* = 9 for 7 consecutive days.

Sample	Nominal IOX concentrations	Recovery (%)	Intraday precision (% CV)	Interday precision (% CV)
Low	25 *μ*g/mL	96 ± 2.5	1.5	3.8
Medium	125 *μ*g/mL	96 ± 1.8	2.5	3.2
High	500 *μ*g/mL	95 ± 2.1	4.0	6.5

**Table 2:** The mean of bodyweight (BW), plasma urea (PU), plasma creatinine concentration (PCr), 7-point reference clearance of IOX (GFR 7 samples: 5, 30, 60, 120, 240, 360, and 480 minutes), and simplified models clearance of IOX in nonazotaemic and azotaemic cats. ^*^Significantly different to nonazotaemic cats at *P* < 0.05.

Cats	BW (kg)	PU (mmol/L)	PCr (*μ*mol/L)	GFR (mL/min/kg) 7 samples	GFR (mL/min/kg) Model A	GFR (mL/min/kg) Model B	GFR (mL/min/kg) Model C	GFR (mL/min/kg) Model D
Nonazotaemic (*n* = 35)	3.5 (2.0–5.0)	15.29 (6.92–24.13)	106 (78–140)	3.40 (1.21–8.62)	3.32 (1.52–6.80)	3.28 (1.64–6.62)	3.08 (1.40–6.87)	3.43 (1.51–8.30)
Azotaemic (*n* = 15)	3.36 (2.0–5.5)	19.78 (16.63–31.38)	160 (143–209)∗	2.38 (1.36–3.47)∗	2.11 (1.25–2.99)∗	2.57 (1.49–4.17)∗	2.28 (1.35–3.66)∗	2.26 (1.45–3.81)∗

**Table 3:** Results of the accuracies of four simplified clearance Models A, B, C, and D in nonazotaemic cats (*n* = 35) and azotaemic cats (*n* = 15) when compared to GFR determination with 7-point reference clearance. There are no significant differences between the different methods neither at the 15%, the 30% levels nor at the 50% level.

Model	Nonazotaemic			Azotaemic		
	Accuracy within			Accuracy within		
	15%	30%	50%	15%	30%	50%
A	87	92	95	92	100	100
B	66	92	95	77	100	100
C	79	92	95	100	100	100
D	47	64	87	53	80	93

## Sources and Manufacturers

Pharsight, Mountain View, CA, USA.
